# Smoking, Alcohol, and Their Interaction in the Risk of Head and Neck Cancer: A Nationwide Cohort Study

**DOI:** 10.1002/cam4.71665

**Published:** 2026-02-20

**Authors:** Eunjung Park, Hee‐Yeon Kang, Yuh‐Seog Jung, Byungmi Kim, Thi Tra Bui, Jin‐Kyoung Oh

**Affiliations:** ^1^ Department of Cancer Control and Population Health National Cancer Center Graduate School of Cancer Science and Policy Goyang Republic of Korea; ^2^ Division of Cancer Prevention National Cancer Control Institute, National Cancer Center Goyang Republic of Korea; ^3^ Department of Otorhinolaryngology–Head and Neck Surgery Center for Thyroid Cancer, National Cancer Center Goyang Republic of Korea

**Keywords:** alcohol drinking, cigarette smoking, cohort study, competing risks, head and neck cancer, interactions

## Abstract

**Background:**

Smoking and alcohol drinking are established causes of head and neck cancer (HNC), yet their combined effect and variation by anatomic sub‐site remain incompletely defined.

**Methods:**

We followed 5,985,244 Korean adults (≥ 30 years) who attended National Health Insurance health screening in 2002–2003 until 31 December 2019. Smoking status (never, former, current) and alcohol intake (none, light, moderate, heavy) were self‐reported at baseline. Incident HNC was ascertained from the national cancer registry. Sub‐distribution hazard ratios (SHRs) were estimated with Fine–Gray competing‐risk regression. Additive interaction was quantified by the relative excess risk due to interaction (RERI), attributable proportion (AP), and synergy index (SI), and multiplicative interaction by a product term.

**Results:**

During 76.5 million person‐years of follow‐up (mean 13‐year follow‐up), 13,491 HNC cases were recorded. Current smoking and heavy alcohol drinking independently increased HNC risk in a dose–response manner. Combined exposure to smoking and heavy drinking showed a supra‐additive risk for HNC overall (SHR, 2.42; 95% CI, 2.28–2.56; RERI, 0.63; AP, 0.26; SI, 1.79), particularly for the oral cavity, oropharynx, nasal cavity/paranasal sinuses, and larynx. Significant multiplicative interactions were also observed in the oral cavity, oropharynx, and hypopharynx.

**Conclusion:**

Concurrent cigarette smoking and heavy alcohol consumption synergistically elevate the risk of HNC across multiple subsites. Integrated public‐health strategies targeting both behaviors are essential to reduce the HNC burden.

**Abbreviations**
AP: attributable proportion due to interaction
BMI: body mass index
CI: confidence interval
HNC: head and neck cancer
HPC: hypopharyngeal cancer
HPV: human papillomavirus
IARC: International Agency for Research on Cancer
LC: laryngeal cancer
NCPSC: nasal cavity and paranasal sinuses cancer
NHIS: National Health Insurance Service
NPC: nasopharyngeal cancer
OCC: oral cavity cancer
OPC: oropharyngeal cancer
RERI: relative excess risk due to interaction
SGC: salivary gland cancer
SHR: subdistribution hazard ratio
SI: synergy index

## 1 Introduction

Head and neck cancer (HNC) encompasses malignancies of the oral cavity, pharynx, larynx, nasal cavity, and related structures and remains a major contributor to global cancer incidence and mortality [1] Cigarette smoking and alcohol consumption are the two most prominent modifiable risk factors for HNC and are each classified as Group I carcinogens by the International Agency for Research on Cancer (IARC) [2].

While the individual effects of smoking and alcohol have been extensively studied, recent attention has increasingly focused on their combined impact. Several studies have suggested that concurrent exposure may lead to a risk that exceeds the sum of their individual contributions. However, evidence on this interaction remains inconsistent, and limited data are available on whether the strength or nature of these associations differs by anatomical subsite within the head and neck region [3, 4]. In addition, many previous studies have been conducted in Western populations, where the patterns of tobacco and alcohol use, as well as the distribution of HNC subsites, may differ from those observed in East Asian countries [5].

Emerging evidence indicates that the carcinogenic synergy between tobacco and alcohol is not homogeneous across head‐and‐neck subsites. Stronger supra‐additive or multiplicative interactions have been reported for the oral cavity, oropharynx, hypopharynx, and larynx, whereas weaker or inconsistent associations are observed for sites such as the salivary glands, nasal cavity, and nasopharynx [4, 6]. These subsite‐specific differences likely reflect variation in mucosal susceptibility, airflow dynamics, and in situ acetaldehyde production within the upper aerodigestive tract [6, 7, 8]. Understanding these patterns is essential because aggregated HNC estimates may obscure clinically meaningful heterogeneity.

Furthermore, investigating combined effects in East Asian populations is particularly important due to distinct genetic and behavioral characteristics. East Asians have a high prevalence of the ALDH2 rs671 variant, which impairs acetaldehyde metabolism and leads to markedly higher acetaldehyde exposure per drink, thereby amplifying alcohol‐related carcinogenicity compared with Western populations [2]. Patterns of tobacco and alcohol use, including higher rates of episodic heavy drinking and differing smoking prevalence, also diverge from those in Western settings [5]. These differences underscore the need for region‐specific evidence on both independent and interactive effects.

Despite this biological and epidemiological rationale, few large‐scale cohort studies in East Asia have comprehensively assessed smoking–alcohol interactions across multiple head‐and‐neck subsites. Prior work has largely relied on Western case–control or pooled analyses, which may not reflect the unique exposure distributions and genetic backgrounds of East Asian populations. Comparative evidence is therefore required to determine whether the interaction patterns observed in Western studies generalize across populations.

To address these limitations, we conducted a population‐based cohort study of nearly 6 million Korean adults, linking national health examination data with cancer registry records. The objective of this study was to comprehensively evaluate the independent and combined associations of cigarette smoking and alcohol consumption with the incidence of HNC overall and by individual anatomical subsites. By identifying variations in risk across subsites, our findings can help inform targeted cancer prevention strategies and enhance understanding of the exposure‐disease relationships underlying HNC.

## 2 Methods

### 2.1 Study Population and Data Sources

We used data from the National Health Insurance Service (NHIS) of South Korea, a universal, mandatory health coverage system comprising approximately 97% of the population, with the remaining 3% covered through medical aid. All beneficiaries are eligible for biennial health screenings, which collect standardized information on sociodemographic factors, lifestyle, and clinical parameters [9]. For this cohort study, we included individuals aged ≥ 30 years who underwent health examinations between 2002 and 2003. These data were linked to the Korea Central Cancer Registry (KCCR) and national death records. Detailed data collection procedures are described elsewhere [10].

**FIGURE 1:**
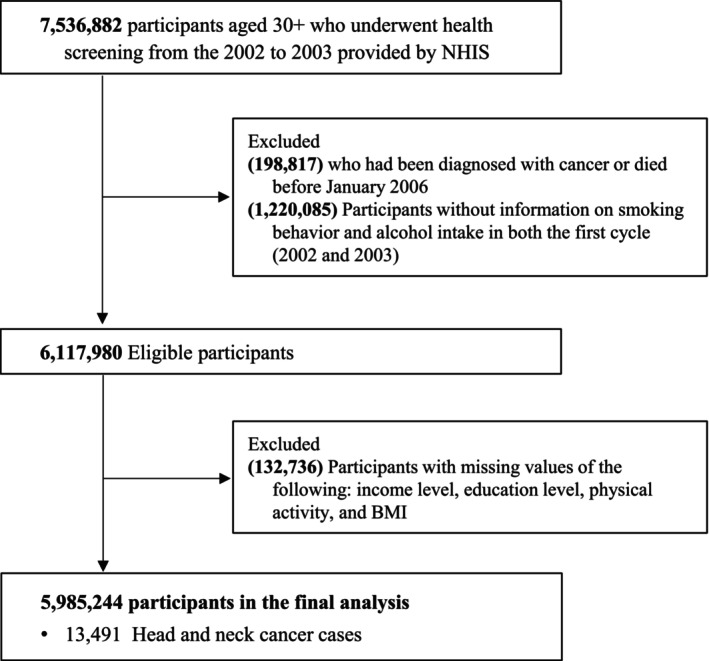
Study population flowchart.

Of 7,536,882 individuals initially screened, we excluded those diagnosed with cancer or who died before the start of follow‐up in 2006 (*n* = 198,817), those with missing information on smoking or alcohol consumption (*n* = 1,220,085), and those missing data on covariates including household income, BMI, or physical activity (*n* = 132,736). The final analytic cohort included 5,985,244 individuals (Figure 1). To reduce potential reverse causation related to the “sick‐quitter” effect, including health‐related former drinkers, baseline health examinations were conducted in 2002–2003, whereas follow‐up for incident HNC began on January 1, 2006. Accordingly, participants who developed any cancer or died between baseline and 2005 were excluded, ensuring a minimum latency of approximately 2–3 years between exposure measurement and outcome ascertainment.

This study was approved by the Institutional Review Board of the National Cancer Center, Korea (approval number: NCC2022‐0180). The study was conducted in accordance with the principles of the Declaration of Helsinki. The requirement for informed consent was waived because anonymised secondary data were used.

### 2.2 Exposure Assessment

Information on cigarette smoking and alcohol consumption was collected via standardized self‐administered questionnaires at baseline. Smoking status was initially categorized as never, former, or current smoker. Because quantitative pack‐year information was available only for current smokers, cumulative exposure for current smokers was categorized into five levels (< 5, 5–< 10, 10–< 20, 20–< 30, or ≥ 30). Alcohol consumption was classified based on estimated ethanol intake, calculated using self‐reported frequency and quantity of alcohol consumed per week, into four categories: non‐drinkers, light (≤ 168 g/week), moderate (> 168 to 336 g/week), and heavy drinkers (> 336 g/week). Accordingly, models evaluating the individual (independent) effects of each exposure used a seven‐category smoking variable (never smoker, former smoker, and five pack‐year categories among current smokers) and a four‐category alcohol variable (non‐, light, moderate, and heavy drinker).

For interaction analyses, smoking was dichotomized as never vs. ever smoker, and alcohol consumption as non‐to‐light vs. moderate‐to‐heavy drinker to ensure adequate cell counts and model stability. Combined exposure was represented using four categories: never smoker and non‐to‐light drinker (reference), never smoker and moderate‐to‐heavy drinker, ever smoker and non‐to‐light drinker, and ever smoker and moderate‐to‐heavy drinker.

### 2.3 Outcome Ascertainment

Incident cases of HNC were identified through the KCCR using the International Classification of Diseases, Tenth Revision codes, C00–C14 and C30–C32. Subsite classification included oral cavity cancer (OCC; C00–C06), salivary gland cancer (SGC; C07–C08), oropharyngeal cancer (OPC; C09–C10), nasopharyngeal cancer (NPC; C11), hypopharyngeal cancer (HPC; C12–C13), nasal cavity and paranasal sinuses cancer (NCPSC; C30–C31), and laryngeal cancer (LC; C32).

### 2.4 Covariates

Covariates were selected a priori based on established literature and a directed acyclic graph representing plausible causal relationships between smoking, alcohol consumption, and HNC. Age (year, continuous), sex, and household income (medical aid or first quartile, second quartile, third quartile, or fourth quartile) were included as major confounders because they act as common determinants of both exposure behaviors and HNC risk. BMI (kg/m^2^, continuous) and physical activity (none, 1–2 days/week, 3–4 days/week, 5–6 days/week, or daily) were adjusted for as potential confounders and prognostic factors, as these lifestyle characteristics are correlated with smoking and alcohol use through shared sociodemographic and behavioral determinants and may independently influence cancer susceptibility. Although data on diet, oral hygiene, occupational factors, and viral infections, including human papillomavirus (HPV) and Epstein–Barr virus (EBV), were unavailable, these factors may act as additional unmeasured risk factors; this potential source of residual confounding is illustrated in the Figure S1.

### 2.5 Statistical Analysis

Follow‐up began on January 1, 2006, and continued until HNC diagnosis, the occurrence of a competing event (other cancers except melanoma or noncancer death), or December 31, 2019, whichever occurred first.

Descriptive statistics were calculated to summarize baseline characteristics of the study population. To evaluate the associations of cigarette smoking and alcohol consumption with the risk of HNC and its anatomical subsites, subdistribution hazard ratios (SHRs) and 95% CIs were estimated using Fine–Gray competing‐risk regression [11] with person‐years as the timescale. We estimated unadjusted SHRs, age‐ and sex‐adjusted SHRs, and multivariable‐adjusted SHRs (fully adjusted models). The fully adjusted models included age, sex, household income, BMI, physical activity, and were mutually adjusted for cigarette smoking and alcohol consumption.

Interaction between smoking and alcohol was evaluated on both additive and multiplicative scales. Additive interaction was quantified using the relative excess risk due to interaction (RERI), attributable proportion (AP), and synergy index (SI) [12], with CIs calculated using the delta method [13]. Multiplicative interaction was assessed via the coefficient of the product term in the Fine–Gray model, interpreted as the ratio of hazard ratios in strata of smoking status (or alcohol consumption). Additionally, absolute interaction cases per 100,000 person‐years were calculated. Further details on interaction metrics are provided in the Supporting Information.

As a sensitivity analysis, we evaluated the independent effects of smoking and alcohol on HNC after restricting cases to squamous cell carcinoma (SCC), defined using ICD‐O‐3 morphology codes (8032, 8033, 8050–8052, 8070–8078, 8082–8084, 8094, 8123). This analysis was conducted to assess whether inclusion of non‐SCC tumors diluted the associations between smoking, alcohol consumption, and HNC risk.

We evaluated model assumptions by introducing time‐varying interaction terms (covariate × log time) in the Fine–Gray models to assess proportional hazards. Multicollinearity was examined using variance inflation factors (VIFs) obtained from a linear regression model including the same set of predictors.

All analyses were performed using SAS Enterprise Guide (PHREG procedure, version 7.1; SAS Institute Inc., Cary, NC, USA). Two‐sided *p*‐values < 0.05 were considered statistically significant. The study was reported following the Strengthening the Reporting of Observational Studies in Epidemiology (STROBE) guideline [14].

## 3 Results

### 3.1 Characteristics of the Study Population

A total of 5,985,244 participants (59.9% men; mean age 47.1 years, standard deviation 11.9) contributed 77,507,093 person‐years of follow‐up. At baseline, 65.9% were never smokers, 3.8% former smokers, and 30.2% current smokers. For alcohol consumption, 54.5% were non‐drinkers, 37.6% light drinkers, 6.3% moderate drinkers, and 1.6% heavy drinkers. Baseline characteristics are summarized in Table 1.

**TABLE 1:** General characteristics of study participants at baseline, 2002–2003.

Characteristic	Participants, no. (%)
Total no.	5,985,244
Person‐years	77,507,093
Age, mean (SD)	47.1 (11.9)
Body mass index, mean (SD)	23.9 (3.0)
Sex	
Men	3,585,999 (59.9)
Women	2,399,245 (40.1)
Smoking status	
Never smokers	3,946,663 (65.9)
Former smokers	229,328 (3.8)
Current smokers	1,809,253 (30.2)
< 5 packyears	294,006 (4.9)
5 to < 10 packyears	276,011 (4.6)
10 to < 20 packyears	727,640 (12.2)
20 to < 30 packyears	317,094 (5.3)
≥ 30 packyears	194,502 (3.3)
Missing	
Alcohol consumption^a^	
Non‐drinkers	3,260,329 (54.5)
Light drinkers	2,252,586 (37.6)
Moderate drinkers	378,162 (6.3)
Heavy drinkers	94,167 (1.6)
Physical activity	
No	3,354,655 (56.1)
1–2 day/week	1,575,501 (26.3)
3–4 day/week	571,335 (9.6)
5–6 day/week	142,771 (2.4)
7 day/week	340,982 (5.7)
Household income	
Medical aid or first quartile	1,084,276 (18.1)
Second quartile	1,247,612 (20.8)
Third quartile	1,642,103 (27.5)
Fourth quartile	2,011,253 (33.6)

Abbreviation: SD, standard deviation.

^a^ Alcohol consumption was categorized as follows: nondrinkers, light drinkers (≤ 168g/week of ethanol), moderate drinkers (〉 168–336g/week), and heavy drinkers (〉336g/week).

During a mean follow‐up of 13 years, 13,491 incident cases of HNC were identified. The most common subsites were the larynx (26.3%) and oral cavity (25.2%), followed by oropharynx (13.6%), nasal cavity/paranasal sinuses (9.5%), nasopharynx (9.2%), salivary glands (8.7%), and hypopharynx (6.7%) (Table 2).

**TABLE 2:** Distribution of head and neck cancer and squamous cell carcinoma (SCC) cases by anatomical subsite (5,985,244 persons, 77,507,093 person‐years).

Classification	ICD‐10 codes	Total cases, *N* (%)	SCC cases, *N* (%)
Head and neck cancer	C00‐C14, C30‐C32	13,491	8870
Oral cavity	C00‐C06	3399 (25.2)	2752 (31.0)
Salivary glands	C07‐C08	1168 (8.7)	65 (0.7)
Oropharyn	C09‐C10	1834 (13.6)	1253 (14.1)
Nasopharynx	C11	1240 (9.2)	475 (5.4)
Hypopharynx	C12‐C13	904 (6.7)	797 (9.0)
Nasal cavity & paranasal sinuses	C30‐C31	1285 (9.5)	383 (4.3)
Larynx	C32	3554 (26.3)	3081 (34.7)
Unknown	C14	107 (0.8)	64 (0.7)

*Note:* SCC classification (ICD‐O‐3 morphology codes 8032, 8033, 8050–8052, 8070–8078, 8082–8084, 8094, 8123) was applied for sensitivity analyses of the independent effects of smoking and alcohol.

Abbreviations: BMI, body mass index; ICD‐10 codes, International Statistical Classification of Diseases and Related Health Problems, Tenth Revision codes; NA, not applicable or not available.

### 3.2 Independent Associations of Smoking and Alcohol With HNC

**FIGURE 2:**
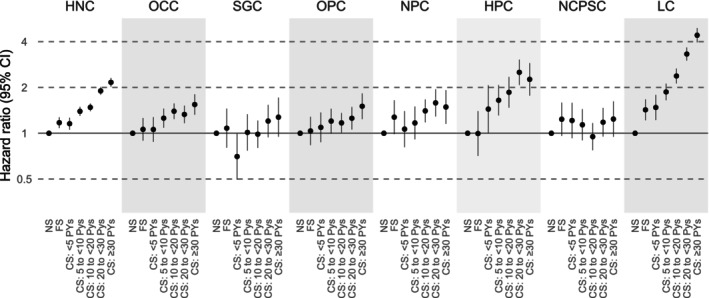
Hazard ratios for head and neck cancer subsites by cigarette smoking. Hazard ratios were adjusted for age, sex, household income, body mass index, physical activity, and alcohol consumption. CS, current smokers; FS, former smokers; HNC, head and neck cancer; HPC, hypopharyngeal cancer; LC, laryngeal cancer; NCPSC, nasal cavity and paranasal sinuses cancer; NPC, nasopharyngeal cancer; NS, never smokers; OCC, oral cavity cancer; OPC, oropharyngeal cancer; PYs, pack‐years; SGC, salivary gland cancer.

In fully adjusted models, current smoking was associated with a higher risk of HNC overall and most subsites, showing clear dose–response trends according to pack‐years. Adjusted SHRs [95% CIs] for current smokers versus never smokers were: HNC, 1.59 [1.53–1.66]; OCC, 1.30 [1.20–1.41]; OPC, 1.21 [1.09–1.35]; NPC, 1.35 [1.19–1.54]; HPC, 1.99 [1.72–2.32]; and LC, 2.68 [2.48–2.90]. No significant associations were observed for SGC (1.04 [0.90–1.21]) or NCPSC (1.09 [0.96–1.25]). Former smoking was modestly associated with HNC and LC (Figure 2, Table S1).

**FIGURE 3:**
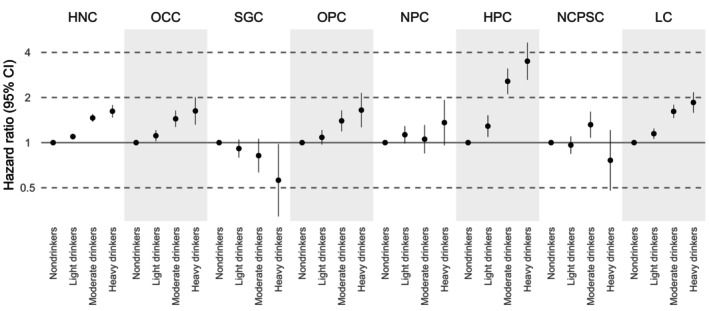
Hazard ratios for head and neck cancer and subsites by alcohol consumption. Hazard ratios were adjusted for age, sex, household income, body mass index, physical activity, and cigarette smoking. Alcohol consumption was categorized as follows: Nondrinkers, light drinkers (≤ 168 g/week of ethanol), moderate drinkers (> 168–336 g/week), and heavy drinkers (> 336 g/week). HNC, head and neck cancer; HPC, hypopharyngeal cancer; LC, laryngeal cancer; OCC, oral cavity cancer; OPC, oropharyngeal cancer; NCPSC, nasal cavity and paranasal sinuses cancer; NPC, nasopharyngeal cancer; SGC, salivary gland cancer.

Heavy alcohol consumption was also associated with increased risk of HNC and several subsites, except SGC and NPC. Compared with non‐drinkers, adjusted SHRs [95% CIs] from fully adjusted models for heavy drinkers were: HNC, 1.62 [1.47–1.78]; OCC, 1.63 [1.32–2.01]; OPC, 1.65 [1.27–2.14]; HPC, 3.49 [2.62–4.65]; and LC, 1.85 [1.58–2.17]. SGC was inversely associated with alcohol consumption (0.56 [0.32–0.98]), and no clear association was observed for NPC (1.36 [0.96–1.93]) or NCPSC (0.76 [0.48–1.21]) (Figure 3, Table S2).

### 3.3 Joint Effects of Smoking and Alcohol Consumption

**FIGURE 4:**
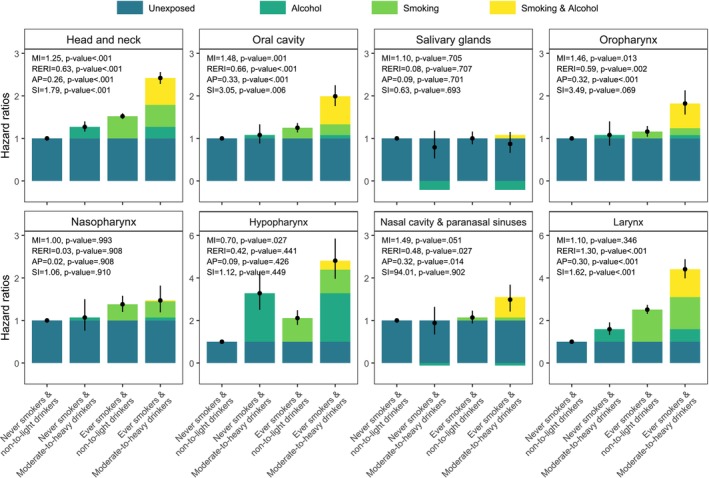
Combined effects of tobacco smoking and alcohol consumption on head and neck cancer and subsites. Hazard ratios were adjusted for age, sex, household income, body mass index, and physical activity. AP, attributable proportion due to interaction; MI, multiplicative interaction; RERI, relative excess risk due to interaction; SI, synergy index.

Participants exposed to both smoking and heavy drinking had substantially elevated risks of HNC and several subsites, compared with those unexposed to either. Adjusted SHRs [95% CIs] from fully adjusted models for ever smokers and moderate‐to‐heavy drinkers, relative to never smokers and non‐to‐light drinkers, were: HNC, 2.42 [2.28–2.56]; OCC, 1.99 [1.76–2.25]; OPC, 1.82 [1.56–2.13]; NPC (1.47 [1.19–1.82]); NCPSC, 1.71 [1.41–2.07]; LC, 7.24 [6.57–7.97]. No excess joint risk was observed for SGC (0.87 [0.66–1.15]) (Figure 4, Table S3).

On the additive scale, combined exposure showed positive interaction for HNC (RERI, 0.63; AP, 0.26; SI, 1.79), OCC (RERI, 0.66; AP, 0.33; SI, 3.05), OPC (RERI, 0.59; AP, 0.32; SI, 3.49), NCPSC (RERI, 0.48; AP, 0.32; SI, 94.01), and LC (RERI, 1.30; AP, 0.30; SI, 1.62). On the multiplicative scale, significant interaction was observed for HNC (ratio of HRs, 1.25; *p* < 0.001), OCC (1.48, *p* = 0.001), and OPC (1.46, *p* = 0.013), whereas no significant multiplicative interaction was found for the other subsites, including LC. Absolute interaction cases per 100,000 person‐years are also reported, providing an absolute‐scale complement to the relative additive interaction measures (Table S3).

### 3.4 Sensitivity Analysis

In a sensitivity analysis restricted to SCC, the associations for cigarette smoking were generally stronger than those observed in the primary analysis, whereas alcohol‐related estimates were largely similar to the main results. However, for NCPSC, alcohol‐related estimates showed a divergent pattern in the SCC‐restricted analysis (Tables S4 and S5).

Model diagnostics showed no major violations of the proportional hazards assumption affecting interpretation of the exposure–outcome associations. Although time‐by‐covariate interaction tests identified statistically significant departures for some covariates, these deviations were minor and expected given the large sample size, and they did not meaningfully influence the estimated subdistribution hazard ratios. No evidence of problematic multicollinearity among covariates was observed, with all VIFs below commonly accepted thresholds (Tables S6 and S7).

## 4 Discussion

In this large population‐based cohort study, both cigarette smoking and alcohol consumption were independently associated with an increased risk of HNC overall and most anatomical subsites. No significant associations were observed for SGC and NCPSC in relation to smoking or for SGC and NPC in relation to alcohol consumption. A dose–response relationship was evident for both exposures. Furthermore, we identified supra‐additive interactions between cigarette smoking and alcohol for HNC and several subsites, including OCC, OPC, NCPSC, and LC, whereas multiplicative interaction was evident for HNC, OCC, and OPC only.

These findings are broadly consistent with previous studies on the association between cigarette smoking and an increased risk of HNC, particularly for HPC and LC [6, 15, 16]. In contrast, associations were weaker or absent for OCC, OPC, and NPC [6]. A meta‐analysis and several cohort studies have reported the larynx and pharynx as the most susceptible sites to the carcinogenic effects of cigarette smoking [4, 6, 7]. This site‐specific difference may be explained by airflow dynamics in the upper aerodigestive tract, where turbulent flow in the larynx leads to prolonged exposure of the laryngeal epithelium to inhaled carcinogens.

The lack of association between cigarette smoking and NCPSC in this study may be due to histologic heterogeneity. Prior research suggests that smoking increases the risk primarily for squamous cell subtypes [16], which comprised only 28.0% NCPSC cases in our cohort. This explanation is further supported by the SCC‐restricted sensitivity analysis, in which smoking‐related estimates for NCPSC were stronger than in the primary analysis, consistent with attenuation of effect estimates when non‐SCC tumors are included. Similarly, the null finding for SGC is consistent with prior studies reporting no association between cigarette smoking and most salivary gland malignancies, with the exception of Warthin tumor [17].

Alcohol consumption was also associated with increased risk of HNC and several subsites, excluding SGC and NPC. This aligns with prior evidence [6, 18] and IARC classification of ethanol and its metabolite acetaldehyde as Group I carcinogens [19]. Acetaldehyde may induce DNA damage and mutations through the formation of DNA adducts [20]. The stronger associations observed for HPC may reflect site‐specific differences in alcohol metabolism and the higher local concentration of salivary acetaldehyde [8, 21, 22]. However, OCC showed a weaker association with alcohol consumption in this study.

The inverse association observed between alcohol consumption and SGC may reflect the distribution of histologic subtypes, such as adenoid cystic or mucoepidermoid carcinoma, which are not known to be alcohol‐related [23]. For NPC, the lack of association contrasts with meta‐analytic findings [24], and may be attributable to methodological differences across studies, including exposure classification and population characteristics.

We observed both supra‐additive and multiplicative interactions between smoking and alcohol for several subsites, including HNC, OCC, and OPC, suggesting synergistic effects. For NCPSC and LC, interactions were observed on the additive scale only, implying excess absolute risk without proportional risk increase. Although LC demonstrated one of the largest additive effects, the multiplicative interaction term was not statistically significant. For HPC, we observed a statistically significant negative interaction on the multiplicative scale despite strong independent effects of smoking and alcohol. Importantly, this finding should not be interpreted as evidence of a true biological antagonism or saturation effect. Several explanations may account for this sub‐additive pattern. One plausible but hypothetical explanation is a ceiling effect at high exposure levels, whereby risk saturation among heavy smokers and drinkers could attenuate the combined effect; however, this could not be formally evaluated in the present analysis. Misclassification of alcohol consumption, particularly underreporting among heavy drinkers, may also bias multiplicative interaction estimates downward. Population‐specific genetic susceptibility—such as the ALDH2 rs671 variant—may additionally modify alcohol‐related carcinogenesis in a non‐linear manner. Residual confounding from behavioral or lifestyle factors correlated with both smoking and alcohol use may also contribute to the attenuation of the multiplicative effect for HPC. In the absence of dose–response modeling using continuous exposure measures, the precise mechanism underlying this negative multiplicative interaction remains uncertain and should be interpreted with caution. These findings are consistent with previous studies [4, 6, 25], and may be explained by alcohol‐induced enhancement of mucosal permeability, facilitating the absorption of tobacco carcinogens [26].

Although previous studies have demonstrated combined effects of smoking and alcohol for pharyngeal cancers, few have disaggregated subsites. Our findings showed interaction for OPC but not for NPC or HPC, despite strong individual associations with both exposures. This discrepancy compared with earlier studies in which pharyngeal cancer sites were grouped together [6, 25] may reflect subsite‐specific differences that are often obscured when these sites are analyzed collectively.

The stronger smoking‐related associations observed in the SCC‐restricted analysis support the interpretation that inclusion of non‐SCC tumors may have attenuated the overall estimates, particularly for subsites with marked histologic heterogeneity. In contrast, the divergent alcohol‐related pattern for NCPSC in the SCC‐restricted analysis likely reflects changes in case composition and limited case numbers, rather than a distinct etiologic effect. Overall, the SCC‐restricted findings support the robustness of the primary results while highlighting potential variation in etiologic relevance across histologic subtypes.

### 4.1 Strengths and Limitations

This study was strengthened by the large population‐based cohort design and the availability of complete follow‐up through linkage with a national cancer registry. Importantly, it provides subsite‐specific risk estimates for HNC, including rarer cancers such as NPC, SGC, and NCPSC, for which evidence remains limited, particularly in East Asian populations.

Several limitations should be considered. First, the study population was restricted to individuals who underwent health examinations, which may introduce selection bias if participants differ systematically from the general population. However, participation rates were relatively high (e.g., 74% in 2019) [27]. Second, alcohol consumption may have been underreported, and we were unable to distinguish lifelong abstainers from former drinkers. Consequently, we could not fully address potential reverse causation arising from individuals who may have reduced or stopped drinking due to underlying health conditions. To mitigate this concern, outcome ascertainment was initiated approximately 2–3 years after baseline exposure assessment, which is expected to reduce the impact of cancers diagnosed shortly after baseline that may reflect disease processes already underway. Third, information on smokeless tobacco use was not collected in the NHIS database. Although smokeless tobacco use was uncommon in South Korea during the baseline period (2002–2003), national surveillance reports have documented increasing use of non‐combustible or non‐cigarette tobacco products since the mid‐2010s, particularly following the introduction of new tobacco products such as snus and nicotine‐containing oral products [28, 29]. Fourth, residual confounding from unmeasured factors, such as diet, oral hygiene, occupational exposures, or viral infections (HPV/EBV) may persist. In South Korea, EBV is a well‐established risk factor for NPC [30], and HPV is implicated in OPC, with increasing relevance in more recent birth cohorts [31, 32]. Because information on EBV and HPV infection status was not available in this study, we could not account for potential variation in viral‐associated carcinogenesis, which may have influenced subsite‐specific risk estimates. This limitation may have led to some attenuation of the observed associations between smoking, alcohol consumption, and cancer risk, particularly for NPC and OPC, rather than an overestimation of these effects. Although the mean baseline age of participants was relatively high (47 years), suggesting a lower prevalence of HPV‐related OPC at cohort entry, this assumption remains uncertain and should be interpreted cautiously, particularly given temporal increases in HPV‐positive OPC during later follow‐up periods. In addition, heavy smokers and drinkers are at increased risk of early death from other causes, such as lung cancer, cardiovascular disease, and liver disease. Although we used Fine–Gray subdistribution hazard models to account for competing risks, informative censoring related to cause‐specific mortality may have slightly attenuated the observed associations. Finally, Multiple statistical tests were performed across overall and subsite‐specific analyses. Multiple testing correction was not applied because the analyses were hypothesis‐driven and limited to predefined anatomical subsites; therefore, findings with borderline significance should be interpreted cautiously.

## 5 Conclusions

In this large nationwide cohort study, cigarette smoking and alcohol consumption were independently associated with an increased risk of HNC overall and across most anatomical subsites. Simultaneous exposure to both cigarette smoking and alcohol consumption further elevated the risk on the additive scale, underscoring the substantial public health impact of combined lifestyle risks. A supra‐additive increase in risk was observed for several subsites, including OCC, OPC, NCPSC, and LC. Importantly, LC demonstrated one of the largest additive excess risks, while the multiplicative interaction term was not statistically significant. These findings support the importance of integrated tobacco and alcohol control strategies for site‐specific HNC prevention.

## Author Contributions

**Eunjung Park:** conceptualization, methodology, data curation, formal analysis, visualization, writing – original draft. **Hee‐Yeon Kang:** formal analysis, writing – review and editing. **Yuh‐Seog Jung:** investigation, writing – review and editing. **Byungmi Kim:** funding acquisition, writing – review and editing. **Thi Tra Bui:** formal analysis, writing – review and editing. **Jin‐Kyoung Oh:** conceptualization, supervision, funding acquisition, Resources, writing – review and editing.

## Funding

This work was supported by a National Cancer Center (Grants NCC‐2210862 and NCC‐2510620) funded by the Korean government, Republic of Korea. The funders had no role in the design or conduct of the study; the collection, management, analysis, and interpretation of the data; the preparation, review, or approval of the manuscript; or the decision to submit the manuscript for publication.

## Ethics Statement

The study protocol was approved by the Institutional Review Board (IRB) of the Korean National Cancer Center institutional review board (IRB No. NCC2022‐0180).

## Consent

The requirement for informed consent was waived because this study used anonymized secondary data.

## Conflicts of Interest

The authors declare no conflicts of interest.

## Supporting information


**Data S1:** cam471665‐sup‐0001‐FigureS1‐TableS1‐S7.docx.

## Data Availability

The data used in this study (NHIS‐2022‐1‐785) were provided by the National Health Insurance Service (NHIS). To protect personal information, the NHIS prohibits transfer, rental, or sale of the database to third parties. Approved researchers can request access through the NHIS website (https://nhiss.nhis.or.kr).
