# Supplementary material for: Smoking, Alcohol, and Their Interaction in the Risk of Head and Neck Cancer: A Nationwide Cohort Study
Source: Cancer Med. 2026 Feb 20;15(3):e71665. doi: 10.1002/cam4.71665 (PMC12927985; doi:10.1002/cam4.71665)
Supplement: Supplementary file 1 — Data S1: cam471665‐sup‐0001‐FigureS1‐TableS1‐S7.docx. [file CAM4-15-e71665-s001.docx]

**Supplementary material**

**Methods** Method for testing interaction on an additive scale

**Fig. S1** Directed acyclic graph (DAG) of assumed causal relationships

**Table S1.** Hazard ratios for head and neck cancer and subsites by cigarette smoking

**Table S2.** Hazard ratios for head and neck cancer and subsites by alcohol consumption

**Table S3.** Hazard ratios and relative and absolute interaction measures for head and neck cancer and subsites by combination of cigarette smoking and alcohol consumption

**Table S4.** Associations of cigarette smoking with head and neck squamous cell carcinoma and subsites

**Table S5.** Associations of alcohol consumption with head and neck squamous cell carcinoma and subsites

Table S6. Assessment of the proportional hazards assumption using time-by-covariate interaction tests

**Table S7**. Variance inflation factors for main exposures and covariates

**Reference**

**Methods**

**Method for testing interaction on an additive scale**

The relative excess risk due to interaction (RERI), attributable proportion (AP), and synergy index (S) were calculated on the additive scale using hazard ratios (HRs) as follows:

$$\mathrm{RERI} =\mathrm{HR}_{A+B+}-\mathrm{HR}_{A+B-}-\mathrm{HR}_{A-B+}+1,$$

$$AP =\frac{\mathrm{HR}_{A+B+}-\mathrm{HR}_{A+B-}-\mathrm{HR}_{A-B+}+1}{\mathrm{HR}_{A+B+}},$$

$$S =\frac{(\mathrm{HR}_{A+B+}-1)}{\left( \mathrm{HR}_{A+B-}-1 \right)+ (\mathrm{HR}_{A-B+}-1)}.$$

A and B denoted the two risk factors being examined, where “+” indicates presence and “–” indicates absence. Positive interaction if RERI>0, AP>0, or S>1; no additive interaction if RERI=0, AP=0, or S=1; and a negative interaction if RERI<0, AP<0, or S<1. RERI measures the excess risk attributable to interaction compared with the sum of the individual effects, whereas AP represents the proportion of the overall effect in the doubly exposed group that is due to interaction. S expresses RERI as a ratio. Because AP and S depend on the magnitude of HRs, RERI often provides more stable and interpretable results (1). These measures assess interaction on the additive scale and complement multiplicative interaction tests.

**Fig. S1**. Directed acyclic graph (DAG) of assumed causal relationships


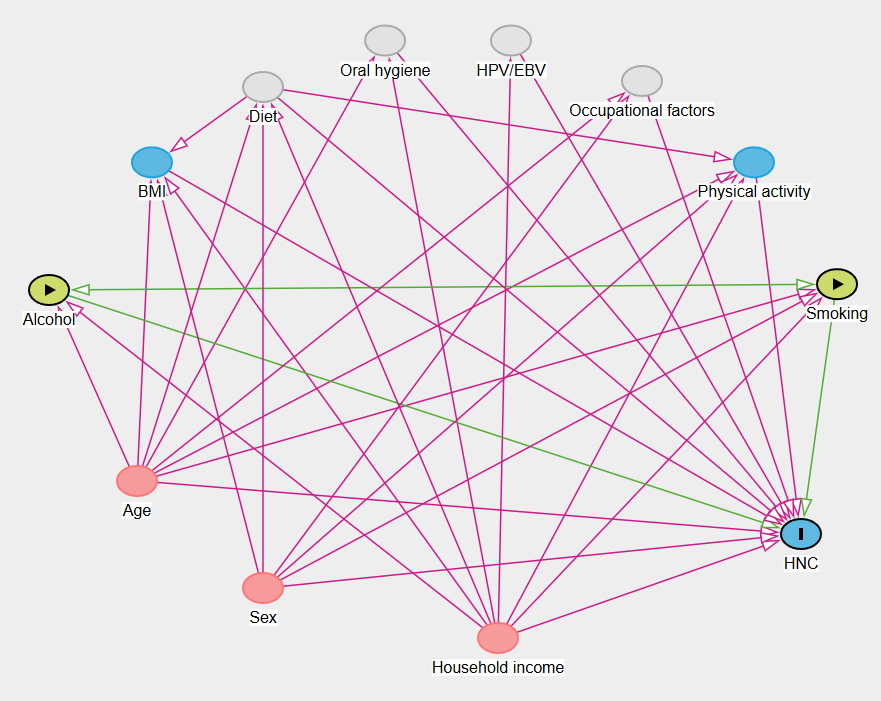

The DAG illustrates the hypothesized relationships among exposures (smoking and alcohol consumption), confounders, and unmeasured risk factors. Age, sex, and household income act as primary confounders influencing both exposure behaviors and HNC risk. BMI and physical activity serve as potential confounders and prognostic factors that reflect correlated lifestyle patterns and may also independently contribute to cancer susceptibility. Diet, oral hygiene, occupational factors, and HPV/EBV infections are included as latent variables representing unmeasured sources of residual confounding.
Minimal sufficient adjustment set (based on the DAG): Age, sex, household income
Expanded adjustment set used in the analysis: Age, sex, household income, BMI, physical activity
The expanded set was selected to reduce residual confounding arising from correlated lifestyle factors while avoiding adjustment for mediators or colliders.

**Table S1.** Hazard ratios for head and neck cancer and subsites by cigarette smoking

| Anatomical sites and smoking status | Participants,  No. | Incident  cases, No. | Person-years | Unadjusted  HR (95% CI) | P-value | Age-sex-adjusted  HR (95% CI) | P-value | Multivariable-adjusted  HR (95% CI) | P-value |
| --- | --- | --- | --- | --- | --- | --- | --- | --- | --- |
| HNC |  |  |  |  |  |  |  |  |  |
| Never smokers | 3,946,663 | 6,678 | 51,040,366 | 1 [Reference] |  | 1 [Reference] |  | 1 [Reference] |  |
| Former smokers | 229,328 | 657 | 2,963,293 | 1.69 (1.56, 1.84) | <.001 | 1.23 (1.13, 1.33) | <.001 | 1.17 (1.08, 1.27) | <.001 |
| Current smokers | 1,809,253 | 6,156 | 23,503,433 | 1.97 (1.90, 2.04) | <.001 | 1.69 (1.63, 1.76) | <.001 | 1.59 (1.53, 1.66) | <.001 |
| <5 packyears | 294,006 | 542 | 3,933,187 | 1.09 (1.00, 1.19) | 0.06 | 1.18 (1.08, 1.29) | <.001 | 1.16 (1.06, 1.27) | 0.002 |
| 5 to <10 packyears | 276,011 | 994 | 3,442,206 | 2.13 (1.99, 2.28) | <.001 | 1.45 (1.35, 1.55) | <.001 | 1.39 (1.30, 1.49) | <.001 |
| 10 to <20 packyears | 727,640 | 1,851 | 9,734,620 | 1.50 (1.43, 1.58) | <.001 | 1.55 (1.47, 1.64) | <.001 | 1.48 (1.40, 1.57) | <.001 |
| 20 to <30 packyears | 317,094 | 1,634 | 3,953,886 | 3.05 (2.89, 3.22) | <.001 | 2.04 (1.93, 2.16) | <.001 | 1.90 (1.79, 2.01) | <.001 |
| ≥30 packyears | 194,502 | 1,135 | 2,439,534 | 3.46 (3.25, 3.68) | <.001 | 2.38 (2.23, 2.54) | <.001 | 2.16 (2.02, 2.31) | <.001 |
| OCC |  |  |  |  |  |  |  |  |  |
| Never smokers | 3,946,663 | 2,007 | 51,040,366 | 1 [Reference] |  | 1 [Reference] |  | 1 [Reference] |  |
| Former smokers | 229,328 | 148 | 2,963,293 | 1.27 (1.07, 1.50) | 0.005 | 1.12 (0.94, 1.33) | 0.20 | 1.06 (0.89, 1.26) | 0.50 |
| Current smokers | 1,809,253 | 1,244 | 23,503,433 | 1.32 (1.23, 1.42) | <.001 | 1.35 (1.25, 1.46) | <.001 | 1.30 (1.20, 1.41) | <.001 |
| <5 packyears | 294,006 | 126 | 3,933,187 | 0.84 (0.70, 1.01) | 0.06 | 1.07 (0.89, 1.29) | 0.48 | 1.06 (0.88, 1.28) | 0.55 |
| 5 to <10 packyears | 276,011 | 217 | 3,442,206 | 1.55 (1.34, 1.78) | <.001 | 1.27 (1.10, 1.47) | 0.001 | 1.26 (1.09, 1.45) | 0.002 |
| 10 to <20 packyears | 727,640 | 432 | 9,734,620 | 1.17 (1.05, 1.30) | 0.004 | 1.45 (1.29, 1.63) | <.001 | 1.40 (1.24, 1.57) | <.001 |
| 20 to <30 packyears | 317,094 | 273 | 3,953,886 | 1.69 (1.49, 1.92) | <.001 | 1.39 (1.22, 1.59) | <.001 | 1.33 (1.17, 1.52) | <.001 |
| ≥30 packyears | 194,502 | 196 | 2,439,534 | 1.98 (1.71, 2.30) | <.001 | 1.67 (1.44, 1.95) | <.001 | 1.55 (1.32, 1.81) | <.001 |
| SGC |  |  |  |  |  |  |  |  |  |
| Never smokers | 3,946,663 | 767 | 51,040,366 | 1 [Reference] |  | 1 [Reference] |  | 1 [Reference] |  |
| Former smokers | 229,328 | 51 | 2,963,293 | 1.14 (0.86, 1.52) | 0.35 | 1.05 (0.78, 1.40) | 0.75 | 1.08 (0.80, 1.45) | 0.61 |
| Current smokers | 1,809,253 | 350 | 23,503,433 | 1.01 (0.90, 1.15) | 0.83 | 1.00 (0.87, 1.15) | >.99 | 1.04 (0.90, 1.21) | 0.56 |
| <5 packyears | 294,006 | 36 | 3,933,187 | 0.63 (0.45, 0.88) | 0.007 | 0.68 (0.48, 0.96) | 0.03 | 0.70 (0.50, 1.00) | 0.05 |
| 5 to <10 packyears | 276,011 | 58 | 3,442,206 | 1.08 (0.83, 1.41) | 0.57 | 0.97 (0.74, 1.28) | 0.84 | 1.01 (0.77, 1.33) | 0.93 |
| 10 to <20 packyears | 727,640 | 126 | 9,734,620 | 0.89 (0.74, 1.08) | 0.23 | 0.94 (0.77, 1.16) | 0.56 | 0.99 (0.80, 1.21) | 0.89 |
| 20 to <30 packyears | 317,094 | 79 | 3,953,886 | 1.28 (1.02, 1.62) | 0.04 | 1.14 (0.89, 1.45) | 0.30 | 1.20 (0.94, 1.54) | 0.14 |
| ≥30 packyears | 194,502 | 51 | 2,439,534 | 1.35 (1.02, 1.79) | 0.04 | 1.19 (0.89, 1.59) | 0.25 | 1.28 (0.95, 1.72) | 0.11 |
| OPC |  |  |  |  |  |  |  |  |  |
| Never smokers | 3,946,663 | 959 | 51,040,366 | 1 [Reference] |  | 1 [Reference] |  | 1 [Reference] |  |
| Former smokers | 229,328 | 96 | 2,963,293 | 1.72 (1.40, 2.13) | <.001 | 1.10 (0.89, 1.36) | 0.40 | 1.04 (0.84, 1.28) | 0.75 |
| Current smokers | 1,809,253 | 779 | 23,503,433 | 1.74 (1.58, 1.91) | <.001 | 1.24 (1.12, 1.38) | <.001 | 1.21 (1.09, 1.35) | <.001 |
| <5 packyears | 294,006 | 92 | 3,933,187 | 1.29 (1.04, 1.60) | 0.02 | 1.09 (0.87, 1.36) | 0.45 | 1.09 (0.87, 1.37) | 0.44 |
| 5 to <10 packyears | 276,011 | 128 | 3,442,206 | 1.91 (1.59, 2.30) | <.001 | 1.19 (0.99, 1.44) | 0.07 | 1.20 (1.00, 1.45) | 0.05 |
| 10 to <20 packyears | 727,640 | 267 | 9,734,620 | 1.51 (1.32, 1.73) | <.001 | 1.20 (1.03, 1.39) | 0.02 | 1.17 (1.01, 1.36) | 0.04 |
| 20 to <30 packyears | 317,094 | 165 | 3,953,886 | 2.14 (1.82, 2.53) | <.001 | 1.28 (1.08, 1.52) | 0.004 | 1.26 (1.06, 1.49) | 0.009 |
| ≥30 packyears | 194,502 | 127 | 2,439,534 | 2.69 (2.24, 3.24) | <.001 | 1.61 (1.33, 1.94) | <.001 | 1.51 (1.24, 1.83) | <.001 |

(continued)

**Table S1.** Hazard ratios for head and neck cancer and subsites by cigarette smoking (continued)

| Anatomical sites and smoking status | Participants,  No. | Incident  cases, No. | Person-years | Unadjusted  HR (95% CI) | P-value | Age-sex-adjusted  HR (95% CI) | P-value | Multivariable-adjusted  HR (95% CI) | P-value |
| --- | --- | --- | --- | --- | --- | --- | --- | --- | --- |
| NPC |  |  |  |  |  |  |  |  |  |
| Never smokers | 3,946,663 | 630 | 51,040,366 | 1 [Reference] |  | 1 [Reference] |  | 1 [Reference] |  |
| Former smokers | 229,328 | 69 | 2,963,293 | 1.89 (1.47, 2.42) | <.0001 | 1.32 (1.02, 1.70) | 0.03 | 1.28 (0.99, 1.65) | 0.06 |
| Current smokers | 1,809,253 | 541 | 23,503,433 | 1.86 (1.66, 2.08) | <.001 | 1.38 (1.22, 1.57) | <.001 | 1.35 (1.19, 1.54) | <.001 |
| <5 packyears | 294,006 | 62 | 3,933,187 | 1.32 (1.02, 1.72) | 0.04 | 1.09 (0.83, 1.42) | 0.55 | 1.07 (0.81, 1.40) | 0.65 |
| 5 to <10 packyears | 276,011 | 74 | 3,442,206 | 1.68 (1.32, 2.14) | <.001 | 1.18 (0.92, 1.50) | 0.20 | 1.17 (0.91, 1.50) | 0.22 |
| 10 to <20 packyears | 727,640 | 212 | 9,734,620 | 1.83 (1.56, 2.13) | <.001 | 1.42 (1.20, 1.69) | <.001 | 1.40 (1.18, 1.67) | <.001 |
| 20 to <30 packyears | 317,094 | 121 | 3,953,886 | 2.39 (1.97, 2.90) | <.001 | 1.61 (1.32, 1.97) | <.001 | 1.58 (1.29, 1.95) | <.001 |
| ≥30 packyears | 194,502 | 72 | 2,439,534 | 2.32 (1.82, 2.96) | <.001 | 1.54 (1.20, 1.98) | <.001 | 1.49 (1.15, 1.92) | 0.002 |
| HPC |  |  |  |  |  |  |  |  |  |
| Never smokers | 3,946,663 | 333 | 51,040,366 | 1 [Reference] |  | 1 [Reference] |  | 1 [Reference] |  |
| Former smokers | 229,328 | 38 | 2,963,293 | 1.96 (1.40, 2.75) | <.001 | 1.11 (0.79, 1.55) | 0.56 | 1.00 (0.71, 1.40) | 0.98 |
| Current smokers | 1,809,253 | 533 | 23,503,433 | 3.36 (2.93, 3.84) | <.001 | 2.60 (2.25, 3.00) | <.001 | 1.99 (1.72, 2.32) | <.001 |
| <5 packyears | 294,006 | 34 | 3,933,187 | 1.37 (0.96, 1.95) | 0.08 | 1.66 (1.16, 2.39) | 0.006 | 1.44 (1.00, 2.07) | 0.05 |
| 5 to <10 packyears | 276,011 | 100 | 3,442,206 | 4.30 (3.44, 5.37) | <.001 | 2.10 (1.67, 2.63) | <.001 | 1.65 (1.31, 2.08) | <.001 |
| 10 to <20 packyears | 727,640 | 122 | 9,734,620 | 1.99 (1.62, 2.45) | <.001 | 2.33 (1.86, 2.94) | <.001 | 1.86 (1.47, 2.35) | <.001 |
| 20 to <30 packyears | 317,094 | 185 | 3,953,886 | 6.92 (5.78, 8.28) | <.001 | 3.45 (2.87, 4.14) | <.001 | 2.52 (2.08, 3.05) | <.001 |
| ≥30 packyears | 194,502 | 92 | 2,439,534 | 5.61 (4.45, 7.06) | <.001 | 3.23 (2.55, 4.08) | <.001 | 2.26 (1.77, 2.89) | <.001 |
| NCPSC |  |  |  |  |  |  |  |  |  |
| Never smokers | 3,946,663 | 785 | 51,040,366 | 1 [Reference] |  | 1 [Reference] |  | 1 [Reference] |  |
| Former smokers | 229,328 | 69 | 2,963,293 | 1.51 (1.18, 1.94) | 0.001 | 1.24 (0.97, 1.60) | 0.09 | 1.24 (0.96, 1.59) | 0.10 |
| Current smokers | 1,809,253 | 431 | 23,503,433 | 1.17 (1.05, 1.32) | 0.007 | 1.11 (0.97, 1.26) | 0.13 | 1.09 (0.96, 1.25) | 0.20 |
| <5 packyears | 294,006 | 62 | 3,933,187 | 1.06 (0.82, 1.37) | 0.66 | 1.20 (0.92, 1.57) | 0.18 | 1.21 (0.93, 1.59) | 0.16 |
| 5 to <10 packyears | 276,011 | 80 | 3,442,206 | 1.46 (1.16, 1.83) | 0.001 | 1.14 (0.90, 1.44) | 0.28 | 1.14 (0.90, 1.44) | 0.29 |
| 10 to <20 packyears | 727,640 | 127 | 9,734,620 | 0.88 (0.73, 1.06) | 0.17 | 0.96 (0.78, 1.18) | 0.68 | 0.95 (0.77, 1.17) | 0.62 |
| 20 to <30 packyears | 317,094 | 99 | 3,953,886 | 1.57 (1.27, 1.94) | <.001 | 1.21 (0.97, 1.50) | 0.09 | 1.18 (0.95, 1.47) | 0.13 |
| ≥30 packyears | 194,502 | 63 | 2,439,534 | 1.63 (1.26, 2.11) | <.001 | 1.27 (0.98, 1.66) | 0.08 | 1.24 (0.95, 1.62) | 0.12 |
| LC |  |  |  |  |  |  |  |  |  |
| Never smokers | 3,946,663 | 1,152 | 51,040,366 | 1 [Reference] |  | 1 [Reference] |  | 1 [Reference] |  |
| Former smokers | 229,328 | 181 | 2,963,293 | 2.70 (2.31, 3.16) | <.001 | 1.50 (1.28, 1.76) | <.001 | 1.43 (1.22, 1.67) | <.001 |
| Current smokers | 1,809,253 | 2,221 | 23,503,433 | 4.11 (3.83, 4.41) | <.001 | 2.96 (2.75, 3.20) | <.001 | 2.68 (2.48, 2.90) | <.001 |
| <5 packyears | 294,006 | 126 | 3,933,187 | 1.47 (1.22, 1.76) | <.001 | 1.55 (1.28, 1.87) | <.001 | 1.48 (1.22, 1.79) | <.001 |
| 5 to <10 packyears | 276,011 | 326 | 3,442,206 | 4.05 (3.58, 4.58) | <.001 | 2.02 (1.78, 2.29) | <.001 | 1.87 (1.65, 2.12) | <.001 |
| 10 to <20 packyears | 727,640 | 550 | 9,734,620 | 2.59 (2.34, 2.87) | <.001 | 2.58 (2.31, 2.89) | <.001 | 2.38 (2.13, 2.67) | <.001 |
| 20 to <30 packyears | 317,094 | 694 | 3,953,886 | 7.51 (6.84, 8.25) | <.001 | 3.71 (3.37, 4.09) | <.001 | 3.32 (3.00, 3.67) | <.001 |
| ≥30 packyears | 194,502 | 525 | 2,439,534 | 9.26 (8.35, 10.27) | <.001 | 5.04 (4.54, 5.61) | <.001 | 4.41 (3.96, 4.92) | <.001 |

The multivariable-adjusted model was considered potential confounders including age, sex, household income, body mass index, physical activity, smoking status, and alcohol consumption, with mutual adjustment for tobacco smoking and alcohol consumption.

Abbreviations: HNC, head and neck cancer; OCC, oral cavity cancer; SGC, salivary gland cancer; OPC, oropharyngeal cancer; NPC, nasopharyngeal cancer; HPC, hypopharyngeal cancer; NCPSC, nasal cavity and paranasal sinuses cancer; and LC, laryngeal cancer.

**Table S2.** Hazard ratios for head and neck cancer and subsites by alcohol consumption

| Anatomical sites and  alcohol consumption | Participants,  No. | Incident  cases, No. | Person-years | Unadjusted  HR (95% CI) | P-value | Age-sex-adjusted  HR (95% CI) | P-value | Multivariable-adjusted  HR (95% CI) | P-value |
| --- | --- | --- | --- | --- | --- | --- | --- | --- | --- |
| HNC |  |  |  |  |  |  |  |  |  |
| Nondrinkers | 3,260,329 | 5,938 | 41,824,796 | 1 [Reference] |  | 1 [Reference] |  | 1 [Reference] |  |
| Light drinkers | 2,252,586 | 5,397 | 29,658,465 | 1.32 (1.27, 1.37) | <.001 | 1.17 (1.12, 1.22) | <.001 | 1.10 (1.05, 1.14) | <.001 |
| Moderate drinkers | 378,162 | 1,674 | 4,834,036 | 2.43 (2.31, 2.57) | <.001 | 1.72 (1.63, 1.82) | <.001 | 1.46 (1.38, 1.55) | <.001 |
| Heavy drinkers | 94,167 | 482 | 1,189,796 | 2.82 (2.57, 3.09) | <.001 | 2.00 (1.82, 2.19) | <.001 | 1.62 (1.47, 1.78) | <.001 |
| OCC |  |  |  |  |  |  |  |  |  |
| Nondrinkers | 3,260,329 | 1,697 | 41,824,796 | 1 [Reference] |  | 1 [Reference] |  | 1 [Reference] |  |
| Light drinkers | 2,252,586 | 1,260 | 29,658,465 | 1.08 (1.00, 1.16) | 0.05 | 1.16 (1.07, 1.26) | <.001 | 1.11 (1.02, 1.21) | 0.01 |
| Moderate drinkers | 378,162 | 344 | 4,834,036 | 1.75 (1.56, 1.96) | <.001 | 1.58 (1.40, 1.79) | <.001 | 1.44 (1.27, 1.63) | <.001 |
| Heavy drinkers | 94,167 | 98 | 1,189,796 | 2.00 (1.63, 2.45) | <.001 | 1.82 (1.47, 2.24) | <.001 | 1.63 (1.32, 2.01) | <.001 |
| SGC |  |  |  |  |  |  |  |  |  |
| Nondrinkers | 3,260,329 | 664 | 41,824,796 | 1 [Reference] |  | 1 [Reference] |  | 1 [Reference] |  |
| Light drinkers | 2,252,586 | 418 | 29,658,465 | 0.91 (0.81, 1.03) | 0.14 | 0.91 (0.79, 1.04) | 0.18 | 0.91 (0.80, 1.05) | 0.20 |
| Moderate drinkers | 378,162 | 73 | 4,834,036 | 0.95 (0.74, 1.21) | 0.66 | 0.85 (0.66, 1.10) | 0.22 | 0.82 (0.63, 1.06) | 0.13 |
| Heavy drinkers | 94,167 | 13 | 1,189,796 | 0.68 (0.39, 1.17) | 0.17 | 0.61 (0.35, 1.06) | 0.08 | 0.56 (0.32, 0.98) | 0.04 |
| OPC |  |  |  |  |  |  |  |  |  |
| Nondrinkers | 3,260,329 | 772 | 41,824,796 | 1 [Reference] |  | 1 [Reference] |  | 1 [Reference] |  |
| Light drinkers | 2,252,586 | 785 | 29,658,465 | 1.47 (1.33, 1.63) | <.001 | 1.13 (1.02, 1.27) | 0.02 | 1.08 (0.97, 1.21) | 0.15 |
| Moderate drinkers | 378,162 | 213 | 4,834,036 | 2.38 (2.04, 2.77) | <.001 | 1.50 (1.28, 1.75) | <.001 | 1.40 (1.19, 1.64) | <.001 |
| Heavy drinkers | 94,167 | 64 | 1,189,796 | 2.88 (2.23, 3.71) | <.001 | 1.80 (1.39, 2.33) | <.001 | 1.65 (1.27, 2.14) | <.001 |
| NPC |  |  |  |  |  |  |  |  |  |
| Nondrinkers | 3,260,329 | 530 | 41,824,796 | 1 [Reference] |  | 1 [Reference] |  | 1 [Reference] |  |
| Light drinkers | 2,252,586 | 565 | 29,658,465 | 1.54 (1.37, 1.74) | <.001 | 1.19 (1.04, 1.35) | 0.009 | 1.13 (0.99, 1.29) | 0.07 |
| Moderate drinkers | 378,162 | 109 | 4,834,036 | 1.77 (1.44, 2.18) | <.001 | 1.18 (0.96, 1.46) | 0.13 | 1.05 (0.85, 1.31) | 0.64 |
| Heavy drinkers | 94,167 | 36 | 1,189,796 | 2.35 (1.68, 3.30) | <.001 | 1.56 (1.11, 2.20) | 0.01 | 1.36 (0.96, 1.93) | 0.08 |
| HPC |  |  |  |  |  |  |  |  |  |
| Nondrinkers | 3,260,329 | 302 | 41,824,796 | 1 [Reference] |  | 1 [Reference] |  | 1 [Reference] |  |
| Light drinkers | 2,252,586 | 350 | 29,658,465 | 1.68 (1.44, 1.96) | <.001 | 1.39 (1.18, 1.64) | <.001 | 1.29 (1.09, 1.52) | 0.003 |
| Moderate drinkers | 378,162 | 188 | 4,834,036 | 5.37 (4.47, 6.44) | <.001 | 3.17 (2.63, 3.82) | <.001 | 2.56 (2.11, 3.12) | <.001 |
| Heavy drinkers | 94,167 | 64 | 1,189,796 | 7.35 (5.61, 9.62) | <.001 | 4.39 (3.34, 5.76) | <.001 | 3.49 (2.62, 4.65) | <.001 |
| NCPSC |  |  |  |  |  |  |  |  |  |
| Nondrinkers | 3,260,329 | 675 | 41,824,796 | 1 [Reference] |  | 1 [Reference] |  | 1 [Reference] |  |
| Light drinkers | 2,252,586 | 462 | 29,658,465 | 0.99 (0.88, 1.12) | 0.88 | 0.98 (0.86, 1.12) | 0.78 | 0.96 (0.84, 1.10) | 0.58 |
| Moderate drinkers | 378,162 | 129 | 4,834,036 | 1.65 (1.37, 1.99) | <.001 | 1.37 (1.12, 1.67) | 0.002 | 1.32 (1.08, 1.61) | 0.007 |
| Heavy drinkers | 94,167 | 19 | 1,189,796 | 0.98 (0.62, 1.54) | 0.91 | 0.81 (0.51, 1.28) | 0.37 | 0.76 (0.48, 1.21) | 0.25 |
| LC |  |  |  |  |  |  |  |  |  |
| Nondrinkers | 3,260,329 | 1,256 | 41,824,796 | 1 [Reference] |  | 1 [Reference] |  | 1 [Reference] |  |
| Light drinkers | 2,252,586 | 1,515 | 29,658,465 | 1.75 (1.62, 1.88) | <.001 | 1.30 (1.20, 1.41) | <.001 | 1.15 (1.06, 1.24) | <.001 |
| Moderate drinkers | 378,162 | 598 | 4,834,036 | 4.11 (3.73, 4.53) | <.001 | 2.23 (2.02, 2.46) | <.001 | 1.62 (1.46, 1.79) | <.001 |
| Heavy drinkers | 94,167 | 185 | 1,189,796 | 5.11 (4.38, 5.96) | <.001 | 2.79 (2.39, 3.26) | <.001 | 1.85 (1.58, 2.17) | <.001 |

The multivariable-adjusted model was considered potential confounders including age, sex, household income, body mass index, physical activity, smoking status, and alcohol consumption, with mutual adjustment for tobacco smoking and alcohol consumption.

Nondrinkers (ethanol of 0g/week), light drinkers (ethanol of ≤168g/week), moderate drinkers (ethanol of <168g to 336g/week), and heavy drinkers (ethanol of >336g/week).

Abbreviations: HNC, head and neck cancer; OCC, oral cavity cancer; SGC, salivary gland cancer; OPC, oropharyngeal cancer; NPC, nasopharyngeal cancer; HPC, hypopharyngeal cancer; NCPSC, nasal cavity and paranasal sinuses cancer; LC, laryngeal cancer.

**Table S3.** Hazard ratios and relative and absolute interaction measures for head and neck cancer and subsites by combination of cigarette smoking and alcohol consumption

| Subsites and smoking status | Alcohol consumption | Participants, No. | Incident  cases, No. | Person-years | aHR (95% CI) | P-value | Measure of effect modification | | | | Absolute interaction cases |
| --- | --- | --- | --- | --- | --- | --- | --- | --- | --- | --- | --- |
|  |  |  |  |  |  |  | Additive scale | | | Multiplicative scale |  |
|  |  |  |  |  |  |  | RERI (95% CI); p-value | AP (95% CI); p-value | SI (95% CI); p-value | Ratio of HRs (95% CI); p-value | per 100,000 person-years |
| HNC |  |  |  |  |  |  |  |  |  |  |  |
| Never smokers | Non-to-light drinkers | 3,806,373 | 6,214 | 49,261,495 | 1 [Reference] |  |  |  |  |  |  |
| Never smokers | Moderate-to-heavy drinkers | 140,290 | 464 | 1,778,872 | 1.27 (1.16, 1.40) | <.001 |  |  |  |  |  |
| Ever smokers | Non-to-light drinkers | 1,706,542 | 5,121 | 22,221,767 | 1.52 (1.46, 1.59) | <.001 |  |  |  |  |  |
| Ever smokers | Moderate-to-heavy drinkers | 332,039 | 1,692 | 4,244,960 | 2.42 (2.28, 2.56) | <.001 | 0.63 (0.46, 0.79); <.001 | 0.26 (0.20, 0.32); <.001 | 1.79 (1.49, 2.15); <.001 | 1.25 (0.11, 0.33); <.001 | 3 |
| OCC |  |  |  |  |  |  |  |  |  |  |  |
| Never smokers | Non-to-light drinkers | 3,806,373 | 1,908 | 49,261,495 |  |  |  |  |  |  |  |
| Never smokers | Moderate-to-heavy drinkers | 140,290 | 99 | 1,778,872 | 1.08 (0.88, 1.33) | 0.47 |  |  |  |  |  |
| Ever smokers | Non-to-light drinkers | 1,706,542 | 1,049 | 22,221,767 | 1.25 (1.14, 1.36) | <.001 |  |  |  |  |  |
| Ever smokers | Moderate-to-heavy drinkers | 332,039 | 343 | 4,244,960 | 1.99 (1.76, 2.25) | <.001 | 0.66 (0.35, 0.97); <.001 | 0.33 (0.20, 0.47); <.001 | 3.05 (1.38, 6.72); 0.006 | 1.48 (0.15, 0.63); 0.001 | 2 |
| SGC |  |  |  |  |  |  |  |  |  |  |  |
| Never smokers | Non-to-light drinkers | 3,806,373 | 741 | 49,261,495 |  |  |  |  |  |  |  |
| Never smokers | Moderate-to-heavy drinkers | 140,290 | 26 | 1,778,872 | 0.79 (0.53, 1.18) | 0.25 |  |  |  |  |  |
| Ever smokers | Non-to-light drinkers | 1,706,542 | 341 | 22,221,767 | 1.00 (0.86, 1.16) | 0.97 |  |  |  |  |  |
| Ever smokers | Moderate-to-heavy drinkers | 332,039 | 60 | 4,244,960 | 0.87 (0.66, 1.15) | 0.32 | 0.08 (-0.32, 0.48); 0.71 | 0.09 (-0.36, 0.54); 0.70 | 0.63 (0.06, 6.22); 0.69 | 1.10 (-0.39, 0.58); 0.71 | 0 |
| OPC |  |  |  |  |  |  |  |  |  |  |  |
| Never smokers | Non-to-light drinkers | 3,806,373 | 896 | 49,261,495 |  |  |  |  |  |  |  |
| Never smokers | Moderate-to-heavy drinkers | 140,290 | 63 | 1,778,872 | 1.08 (0.83, 1.40) | 0.56 |  |  |  |  |  |
| Ever smokers | Non-to-light drinkers | 1,706,542 | 661 | 22,221,767 | 1.16 (1.04, 1.29) | 0.01 |  |  |  |  |  |
| Ever smokers | Moderate-to-heavy drinkers | 332,039 | 214 | 4,244,960 | 1.82 (1.56, 2.13) | <.001 | 0.59 (0.21, 0.96); 0.002 | 0.32 (0.14, 0.51); 0.001 | 3.49 (0.91, 13.44); 0.07 | 1.46 (0.08, 0.68); 0.01 | 0 |
| NPC |  |  |  |  |  |  |  |  |  |  |  |
| Never smokers | Non-to-light drinkers | 3,806,373 | 594 | 49,261,495 |  |  |  |  |  |  |  |
| Never smokers | Moderate-to-heavy drinkers | 140,290 | 36 | 1,778,872 | 1.07 (0.76, 1.50) | 0.71 |  |  |  |  |  |
| Ever smokers | Non-to-light drinkers | 1,706,542 | 501 | 22,221,767 | 1.38 (1.20, 1.58) | <.001 |  |  |  |  |  |
| Ever smokers | Moderate-to-heavy drinkers | 332,039 | 109 | 4,244,960 | 1.47 (1.19, 1.82) | <.001 | 0.03 (-0.44, 0.50); 0.91 | 0.02 (-0.30, 0.34); 0.91 | 1.06 (0.37, 3.02); 0.91 | 1.00 (-0.40, 0.40); >.99 | -1 |
| HPC |  |  |  |  |  |  |  |  |  |  |  |
| Never smokers | Non-to-light drinkers | 3,806,373 | 266 | 49,261,495 |  |  |  |  |  |  |  |
| Never smokers | Moderate-to-heavy drinkers | 140,290 | 67 | 1,778,872 | 3.28 (2.50, 4.30) | <.001 |  |  |  |  |  |
| Ever smokers | Non-to-light drinkers | 1,706,542 | 386 | 22,221,767 | 2.11 (1.79, 2.48) | <.001 |  |  |  |  |  |
| Ever smokers | Moderate-to-heavy drinkers | 332,039 | 185 | 4,244,960 | 4.81 (3.96, 5.85) | <.001 | 0.42 (-0.65, 1.50); 0.44 | 0.09 (-0.13, 0.30); 0.43 | 1.12 (0.83, 1.52); 0.45 | 0.70 (-0.68, -0.04); 0.03 | -1 |
| NCPSC |  |  |  |  |  |  |  |  |  |  |  |
| Never smokers | Non-to-light drinkers | 3,806,373 | 749 | 49,261,495 | 1 [Reference] |  |  |  |  |  |  |
| Never smokers | Moderate-to-heavy drinkers | 140,290 | 36 | 1,778,872 | 1.30 (0.93, 1.82) | 0.12 |  |  |  |  |  |
| Ever smokers | Non-to-light drinkers | 1,706,542 | 388 | 22,221,767 | 1.13 (1.00, 1.27) | 0.05 |  |  |  |  |  |
| Ever smokers | Moderate-to-heavy drinkers | 332,039 | 112 | 4,244,960 | 1.71 (1.41, 2.07) | <.001 | 0.48 (0.06, 0.91); 0.03 | 0.32 (0.07, 0.58); 0.01 | 94.01 (0.00, Inf); 0.90 | 1.49 (0.00, 0.80); 0.05 | 0 |
| LC |  |  |  |  |  |  |  |  |  |  |  |
| Never smokers | Non-to-light drinkers | 3,806,373 | 1,020 | 49,261,495 | 1 [Reference] |  |  |  |  |  |  |
| Never smokers | Moderate-to-heavy drinkers | 140,290 | 132 | 1,778,872 | 3.51 (2.93, 4.21) | <.001 |  |  |  |  |  |
| Ever smokers | Non-to-light drinkers | 1,706,542 | 1,751 | 22,221,767 | 3.76 (3.49, 4.06) | <.001 |  |  |  |  |  |
| Ever smokers | Moderate-to-heavy drinkers | 332,039 | 651 | 4,244,960 | 7.24 (6.57, 7.97) | <.001 | 1.30 (0.85, 1.75); <.001 | 0.30 (0.21, 0.38); <.001 | 1.62 (1.36, 1.93); <.001 | 1.10 (-0.11, 0.30); 0.35 | 2 |

Additive interaction was assessed using the relative excess risk due to interaction (RERI), attributable proportion due to interaction (AP), and synergy index (S). The measure of interaction on a multiplicative scale, the ratio of HRs in strata of smoking status (or alcohol consumption). Absolute interaction cases represent the excess number of cases per 100,000 person-years attributable specifically to the interaction between smoking and alcohol, beyond the sum of their individual effects. Absolute interaction cases were calculated as: $IR_{A+B+}-IR_{A+B-}-IR_{A-B+}+IR_{A-B-}$, where IR denotes incidence rate per 100,000 person-years, A indicates smoking (ever vs. never), and B indicates alcohol consumption (moderate-to-heavy vs. non-to-light). Ever smokers were individuals who have smoked either currently or in the past. Nondrinkers were defined as ethanol intake of 0 g/week; light drinkers as ≤168 g/week; moderate drinkers as >168 to 336 g/week; and heavy drinkers as >336 g/week.

Abbreviations: HNC, head and neck cancer; OCC, oral cavity cancer; SGC, salivary gland cancer; OPC, oropharyngeal cancer; NPC, nasopharyngeal cancer; HPC, hypopharyngeal cancer; NCPSC, nasal cavity and paranasal sinuses cancer; LC, laryngeal cancer; RERI, relative excess risk due to interaction; AP, attributable proportion due to interaction; and SI, synergy index

**Table S4.** Associations of cigarette smoking with head and neck squamous cell carcinoma and subsites (continued)

|  | Total head and neck cancer |  | Squamous cell carcinoma |  |
| --- | --- | --- | --- | --- |
| Anatomical sites and smoking status | Adjusted HR (95% CI) | P-value | Adjusted HR (95% CI) | P-value |
| HNC |  |  |  |  |
| Never smokers | 1 [Reference] |  | 1 [Reference] |  |
| Former smokers | 1.17 (1.08, 1.27) | <.001 | 1.21 (1.12, 1.30) | <.001 |
| Current smokers, <5 packyears | 1.16 (1.06, 1.27) | 0.002 | 1.23 (1.10, 1.37) | <.001 |
| Current smokers, 5 to <10 packyears | 1.39 (1.30, 1.49) | <.001 | 1.45 (1.33, 1.58) | <.001 |
| Current smokers, 10 to <20 packyears | 1.48 (1.40, 1.57) | <.001 | 1.91 (1.79, 2.05) | <.001 |
| Current smokers, 20 to <30 packyears | 1.90 (1.79, 2.01) | <.001 | 2.25 (2.10, 2.42) | <.001 |
| Current smokers, ≥30 packyears | 2.16 (2.02, 2.31) | <.001 | 2.81 (2.60, 3.04) | <.001 |
| OCC |  |  |  |  |
| Never smokers | 1 [Reference] |  | 1 [Reference] |  |
| Former smokers | 1.06 (0.89, 1.26) | 0.50 | 0.99 (0.87, 1.13) | 0.88 |
| Current smokers, <5 packyears | 1.06 (0.88, 1.28) | 0.55 | 1.04 (0.86, 1.26) | 0.69 |
| Current smokers, 5 to <10 packyears | 1.26 (1.09, 1.45) | 0.002 | 1.17 (1.00, 1.37) | 0.06 |
| Current smokers, 10 to <20 packyears | 1.40 (1.24, 1.57) | <.001 | 1.52 (1.34, 1.72) | <.001 |
| Current smokers, 20 to <30 packyears | 1.33 (1.17, 1.52) | <.001 | 1.57 (1.36, 1.81) | <.001 |
| Current smokers, ≥30 packyears | 1.55 (1.32, 1.81) | <.001 | 1.74 (1.47, 2.06) | <.001 |
| SGC |  |  |  |  |
| Never smokers | 1 [Reference] |  | 1 [Reference] |  |
| Former smokers | 1.08 (0.80, 1.45) | 0.61 | 2.19 (1.06, 4.54) | 0.04 |
| Current smokers, <5 packyears | 0.70 (0.50, 1.00) | 0.05 | 0.94 (0.22, 4.03) | 0.94 |
| Current smokers, 5 to <10 packyears | 1.01 (0.77, 1.33) | 0.93 | 0.91 (0.27, 3.08) | 0.88 |
| Current smokers, 10 to <20 packyears | 0.99 (0.80, 1.21) | 0.89 | 1.68 (0.73, 3.87) | 0.22 |
| Current smokers, 20 to <30 packyears | 1.20 (0.94, 1.54) | 0.14 | 1.44 (0.54, 3.83) | 0.47 |
| Current smokers, ≥30 packyears | 1.28 (0.95, 1.72) | 0.11 | 2.01 (0.67, 6.03) | 0.22 |
| OPC |  |  |  |  |
| Never smokers | 1 [Reference] |  | 1 [Reference] |  |
| Former smokers | 1.04 (0.84, 1.28) | 0.75 | 1.18 (1.00, 1.40) | 0.05 |
| Current smokers, <5 packyears | 1.09 (0.87, 1.37) | 0.44 | 1.19 (0.92, 1.54) | 0.19 |
| Current smokers, 5 to <10 packyears | 1.20 (1.00, 1.45) | 0.05 | 1.05 (0.83, 1.33) | 0.68 |
| Current smokers, 10 to <20 packyears | 1.17 (1.01, 1.36) | 0.04 | 1.44 (1.21, 1.71) | <.001 |
| Current smokers, 20 to <30 packyears | 1.26 (1.06, 1.49) | 0.009 | 1.25 (1.01, 1.54) | 0.04 |
| Current smokers, ≥30 packyears | 1.51 (1.24, 1.83) | <.001 | 2.10 (1.71, 2.58) | <.001 |
| NPC |  |  |  |  |
| Never smokers | 1 [Reference] |  | 1 [Reference] |  |
| Former smokers | 1.28 (0.99, 1.65) | 0.06 | 0.85 (0.62, 1.16) | 0.31 |
| Current smokers, <5 packyears | 1.07 (0.81, 1.40) | 0.65 | 0.99 (0.67, 1.47) | 0.96 |
| Current smokers, 5 to <10 packyears | 1.17 (0.91, 1.50) | 0.22 | 0.99 (0.68, 1.44) | 0.95 |
| Current smokers, 10 to <20 packyears | 1.40 (1.18, 1.67) | <.001 | 1.44 (1.11, 1.88) | 0.007 |
| Current smokers, 20 to <30 packyears | 1.58 (1.29, 1.95) | <.001 | 1.70 (1.24, 2.33) | <.001 |
| Current smokers, ≥30 packyears | 1.49 (1.15, 1.92) | 0.002 | 1.63 (1.09, 2.44) | 0.02 |

The multivariable-adjusted model was considered potential confounders including age, sex, household income, body mass index, physical activity, smoking status, and alcohol consumption, with mutual adjustment for tobacco smoking and alcohol consumption.

Abbreviations: HNC, head and neck cancer; OCC, oral cavity cancer; SGC, salivary gland cancer; OPC, oropharyngeal cancer; NPC, nasopharyngeal cancer; HPC, hypopharyngeal cancer; NCPSC, nasal cavity and paranasal sinuses cancer; and LC, laryngeal cancer.

**Table S4.** Associations of cigarette smoking with head and neck squamous cell carcinoma and subsites

|  | Total head and neck cancer |  | Squamous cell carcinoma |  |
| --- | --- | --- | --- | --- |
| Anatomical sites and smoking status | Adjusted HR (95% CI) | P-value | Adjusted HR (95% CI) | P-value |
| HPC |  |  |  |  |
| Never smokers | 1 [Reference] |  | 1 [Reference] |  |
| Former smokers | 1.00 (0.71, 1.40) | 0.98 | 1.13 (0.79, 1.61) | 0.50 |
| Current smokers, <5 packyears | 1.44 (1.00, 2.07) | 0.05 | 1.21 (0.72, 2.05) | 0.47 |
| Current smokers, 5 to <10 packyears | 1.65 (1.31, 2.08) | <.001 | 1.41 (0.94, 2.11) | 0.10 |
| Current smokers, 10 to <20 packyears | 1.86 (1.47, 2.35) | <.001 | 1.98 (1.43, 2.73) | <.001 |
| Current smokers, 20 to <30 packyears | 2.52 (2.08, 3.05) | <.001 | 1.83 (1.27, 2.64) | 0.001 |
| Current smokers, ≥30 packyears | 2.26 (1.77, 2.89) | <.001 | 2.34 (1.54, 3.55) | <.001 |
| NCPSC |  |  |  |  |
| Never smokers | 1 [Reference] |  | 1 [Reference] |  |
| Former smokers | 1.24 (0.96, 1.59) | 0.10 | 1.17 (0.91, 1.50) | 0.23 |
| Current smokers, <5 packyears | 1.21 (0.93, 1.59) | 0.16 | 1.18 (0.76, 1.83) | 0.45 |
| Current smokers, 5 to <10 packyears | 1.14 (0.90, 1.44) | 0.29 | 1.63 (1.25, 2.13) | <.001 |
| Current smokers, 10 to <20 packyears | 0.95 (0.77, 1.17) | 0.62 | 2.24 (1.75, 2.86) | <.001 |
| Current smokers, 20 to <30 packyears | 1.18 (0.95, 1.47) | 0.13 | 2.64 (2.11, 3.29) | <.001 |
| Current smokers, ≥30 packyears | 1.24 (0.95, 1.62) | 0.12 | 2.81 (2.17, 3.63) | <.001 |
| LC |  |  |  |  |
| Never smokers | 1 [Reference] |  | 1 [Reference] |  |
| Former smokers | 1.43 (1.22, 1.67) | <.001 | 1.56 (1.38, 1.77) | <.001 |
| Current smokers, <5 packyears | 1.48 (1.22, 1.79) | <.001 | 1.52 (1.24, 1.87) | <.001 |
| Current smokers, 5 to <10 packyears | 1.87 (1.65, 2.12) | <.001 | 2.03 (1.76, 2.34) | <.001 |
| Current smokers, 10 to <20 packyears | 2.38 (2.13, 2.67) | <.001 | 2.86 (2.53, 3.24) | <.001 |
| Current smokers, 20 to <30 packyears | 3.32 (3.00, 3.67) | <.001 | 3.65 (3.25, 4.10) | <.001 |
| Current smokers, ≥30 packyears | 4.41 (3.96, 4.92) | <.001 | 5.01 (4.42, 5.68) | <.001 |

The multivariable-adjusted model was considered potential confounders including age, sex, household income, body mass index, physical activity, smoking status, and alcohol consumption, with mutual adjustment for tobacco smoking and alcohol consumption.

Abbreviations: HNC, head and neck cancer; OCC, oral cavity cancer; SGC, salivary gland cancer; OPC, oropharyngeal cancer; NPC, nasopharyngeal cancer; HPC, hypopharyngeal cancer; NCPSC, nasal cavity and paranasal sinuses cancer; and LC, laryngeal cancer.

**Table S5.** Associations of alcohol consumption with head and neck squamous cell carcinoma and subsites

|  | Total head and neck cancer |  | Squamous cell carcinoma |  |
| --- | --- | --- | --- | --- |
| Anatomical sites and alcohol consumption | Adjusted HR (95% CI) | P-value | Adjusted HR (95% CI) | P-value |
| HNC |  |  |  |  |
| Nondrinkers | 1 [Reference] |  | 1 [Reference] |  |
| Light drinkers | 1.10 (1.05, 1.14) | <.001 | 1.16 (1.10, 1.23) | <.001 |
| Moderate drinkers | 1.46 (1.38, 1.55) | <.001 | 1.54 (1.43, 1.65) | <.001 |
| Heavy drinkers | 1.62 (1.47, 1.78) | <.001 | 1.97 (1.82, 2.14) | <.001 |
| OCC |  |  |  |  |
| Nondrinkers | 1 [Reference] |  | 1 [Reference] |  |
| Light drinkers | 1.11 (1.02, 1.21) | 0.01 | 1.19 (1.09, 1.31) | <.001 |
| Moderate drinkers | 1.44 (1.27, 1.63) | <.001 | 1.47 (1.29, 1.67) | <.001 |
| Heavy drinkers | 1.63 (1.32, 2.01) | <.001 | 1.75 (1.49, 2.06) | <.001 |
| SGC |  |  |  |  |
| Nondrinkers | 1 [Reference] |  | 1 [Reference] |  |
| Light drinkers | 0.91 (0.80, 1.05) | 0.20 | 0.72 (0.40, 1.27) | 0.25 |
| Moderate drinkers | 0.82 (0.63, 1.06) | 0.13 | 0.50 (0.17, 1.43) | 0.19 |
| Heavy drinkers | 0.56 (0.32, 0.98) | 0.04 | 0.94 (0.34, 2.64) | 0.91 |
| OPC |  |  |  |  |
| Nondrinkers | 1 [Reference] |  | 1 [Reference] |  |
| Light drinkers | 1.08 (0.97, 1.21) | 0.15 | 1.12 (0.97, 1.28) | 0.12 |
| Moderate drinkers | 1.40 (1.19, 1.64) | <.001 | 1.54 (1.29, 1.84) | <.001 |
| Heavy drinkers | 1.65 (1.27, 2.14) | <.001 | 1.72 (1.38, 2.15) | <.001 |
| NPC |  |  |  |  |
| Nondrinkers | 1 [Reference] |  | 1 [Reference] |  |
| Light drinkers | 1.13 (0.99, 1.29) | 0.07 | 1.15 (0.93, 1.42) | 0.20 |
| Moderate drinkers | 1.05 (0.85, 1.31) | 0.64 | 0.93 (0.66, 1.31) | 0.67 |
| Heavy drinkers | 1.36 (0.96, 1.93) | 0.08 | 1.08 (0.70, 1.67) | 0.73 |
| HPC |  |  |  |  |
| Nondrinkers | 1 [Reference] |  | 1 [Reference] |  |
| Light drinkers | 1.29 (1.09, 1.52) | 0.003 | 1.30 (1.07, 1.58) | 0.009 |
| Moderate drinkers | 2.56 (2.11, 3.12) | <.001 | 2.62 (2.10, 3.26) | <.001 |
| Heavy drinkers | 3.49 (2.62, 4.65) | <.001 | 4.02 (3.19, 5.05) | <.001 |
| NCPSC |  |  |  |  |
| Nondrinkers | 1 [Reference] |  | 1 [Reference] |  |
| Light drinkers | 0.96 (0.84, 1.10) | 0.58 | 1.11 (0.86, 1.43) | 0.43 |
| Moderate drinkers | 1.32 (1.08, 1.61) | 0.007 | 1.39 (0.98, 1.97) | 0.06 |
| Heavy drinkers | 0.76 (0.48, 1.21) | 0.25 | 1.82 (1.21, 2.74) | 0.004 |
| LC |  |  |  |  |
| Nondrinkers | 1 [Reference] |  | 1 [Reference] |  |
| Light drinkers | 1.15 (1.06, 1.24) | <.001 | 1.14 (1.04, 1.25) | 0.006 |
| Moderate drinkers | 1.62 (1.46, 1.79) | <.001 | 1.47 (1.31, 1.65) | <.001 |
| Heavy drinkers | 1.85 (1.58, 2.17) | <.001 | 1.90 (1.67, 2.15) | <.001 |

The multivariable-adjusted model was considered potential confounders including age, sex, household income, body mass index, physical activity, smoking status, and alcohol consumption, with mutual adjustment for tobacco smoking and alcohol consumption.

Nondrinkers (ethanol of 0g/week), light drinkers (ethanol of ≤168g/week), moderate drinkers (ethanol of <168g to 336g/week), and heavy drinkers (ethanol of >336g/week).

Abbreviations: HNC, head and neck cancer; OCC, oral cavity cancer; SGC, salivary gland cancer; OPC, oropharyngeal cancer; NPC, nasopharyngeal cancer; HPC, hypopharyngeal cancer; NCPSC, nasal cavity and paranasal sinuses cancer; LC, laryngeal cancer.

**Table S6.** Assessment of the proportional hazards assumption using time-by-covariate interaction tests

| Subsite | Exposures | p-value |
| --- | --- | --- |
| HNC | Smoking status | 0.011 |
|  | Alcohol consumption | 0.017 |
|  | Age | 0.004 |
|  | Sex | 0.007 |
|  | Household income | 0.013 |
|  | Physical activity | 0.047 |
|  | Body mass index | 0.005 |
| OCC | Smoking status | 0.044 |
|  | Alcohol consumption | 0.033 |
| SGC | Smoking status | 0.812 |
|  | Alcohol consumption | 0.043 |
| OPC | Smoking status | 0.028 |
|  | Alcohol consumption | 0.015 |
| NPC | Smoking status | 0.043 |
|  | Alcohol consumption | 0.014 |
| HPC | Smoking status | 0.090 |
|  | Alcohol consumption | 0.020 |
| NCP | Smoking status | 0.118 |
|  | Alcohol consumption | 0.037 |
| LC | Smoking status | 0.025 |
|  | Alcohol consumption | 0.017 |

Note: The proportional hazards assumption was evaluated using interaction terms between covariates and log-transformed follow-up time. Given the large sample size, these tests are highly sensitive and may detect minor time-varying effects

Abbreviations: HNC, head and neck cancer; OCC, oral cavity cancer; SGC, salivary gland cancer; OPC, oropharyngeal cancer; NPC, nasopharyngeal cancer; HPC, hypopharyngeal cancer; NCPSC, nasal cavity and paranasal sinuses cancer; LC, laryngeal cancer.

**Table S7**. Variance inflation factors for main exposures and covariates

| Variable |  | Variance inflation factors |
| --- | --- | --- |
| Smoking status | Never | ‒ |
|  | Former | 1.27 |
|  | Current, <5 packyears | 1.19 |
|  | Current, 5 to <10 packyears | 1.19 |
|  | Current, 10 to <20 packyears | 1.38 |
|  | Current, 20 to <30 packyears | 1.17 |
|  | Current, 30+ packyears | 1.12 |
| Alcohol consumption | Non | ‒ |
|  | Light | 1.44 |
|  | Moderate | 1.23 |
|  | Heavy | 1.12 |
| Combination of smoking and alcohol | Never smoker & non-to-light | ‒ |
|  | Never smoker & moderate-to-heavy drinker | 1.06 |
|  | Ever smoker & non-to-light | 1.63 |
|  | Ever smoker & moderate-to-heavy drinker | 1.24 |
| Age |  | 1.21 |
| Sex | Men | ‒ |
|  | Women | 1.74 |
| Household income | Medical aid or first quartile | ‒ |
|  | Second quartile | 1.69 |
|  | Third quartile | 1.76 |
|  | Fourth quartile | 1.87 |
| BMI |  | 1.07 |
| Physical activity | No | ‒ |
|  | 1-2 d/wk | 1.16 |
|  | 3-4 d/wk | 1.09 |
|  | 5-6 d/wk | 1.02 |
|  | 7 d/wk | 1.05 |

Note: Multicollinearity was assessed using variance inflation factors, with all values below commonly used thresholds.

**Reference**

1. Knol MJ, VanderWeele TJ. Recommendations for presenting analyses of effect modification and interaction. International journal of epidemiology. 2012;41(2):514-20.
